# Numerical Investigation on the Role of Mechanical Factors Contributing to Globe Flattening in States of Elevated Intracranial Pressure

**DOI:** 10.3390/life10120316

**Published:** 2020-11-28

**Authors:** Jafar A. Mehr, Heather E. Moss, Hamed Hatami-Marbini

**Affiliations:** 1 Computational Biomechanics Research Laboratory, Mechanical and Industrial Engineering Department, University of Illinois at Chicago, Chicago, IL 60612, USA; jarash2@uic.edu; 2 Departments of Ophthalmology and Neurology & Neurosciences, Stanford University, Palo Alto, CA 94303, USA; hemoss@stanford.edu

**Keywords:** posterior globe, numerical analysis, material properties, MR images

## Abstract

Flattening of the posterior eye globe in the magnetic resonance (MR) images is a sign associated with elevated intracranial pressure (ICP), often seen in people with idiopathic intracranial hypertension (IIH). The exact underlying mechanisms of globe flattening (GF) are not fully known but mechanical factors are believed to play a role. In the present study, we investigated the effects of material properties and pressure loads on GF. For this purpose, we used a generic finite element model to investigate the deformation of the posterior eyeball. The degree of GF in numerical models and the significance of different mechanical factors on GF were characterized using an automated angle-slope technique and a statistical measure. From the numerical models, we found that ICP had the most important role in GF. We also showed that the angle-slope graphs pertaining to MR images from five people with high ICP can be represented numerically by manipulating the parameters of the finite element model. This numerical study suggests that GF observed in IIH patients can be accounted for by the forces caused by elevation of ICP from its normal level, while material properties of ocular tissues, such as sclera (SC), peripapillary sclera (PSC), and optic nerve (ON), would impact its severity.

## 1. Introduction

Idiopathic intracranial hypertension (IIH) is a state of increased intracranial pressure (ICP), first described by Quinke in 1893, with diagnostic criteria based on the description by Walter Dandy in 1937 [1]. It usually occurs in obese but otherwise healthy women of childbearing age, causing headaches and pulsatile tinnitus [2]. Visual impairment, sometimes leading to blindness, is related to retinal ganglion cell dysfunction in the setting of papilledema, which is swelling of the optic nerve head (ONH) caused by high cerebrospinal fluid (CSF) pressure in the optic nerve sheath (ONS). Although a connection between IIH and obesity has been recognized [3], the reasons behind this disease are still not fully understood. Hence, there is no causally directed treatment. Instead, medical and surgical treatments aimed at normalization of ICP are used to control symptoms and limit vision loss.

High ICP is associated with structural ophthalmic changes including flattening of the posterior globe and inward protrusion of the ONH, both visible on magnetic resonance (MR) images of the eyes [4]. Expansion of the subarachnoid space (SAS) inside the ONS behind the eye is another sign in the MR images in the elevated ICP-related diseases, including IIH and diseases causing secondary intracranial hypertension (IH) such as venous sinus thrombosis and brain tumors [5,6,7]. In addition to MR images, the standardized ultrasonography can be used for measuring the ONS diameter and characterizing the expansion of the SAS inside the ONS because of the increase of subarachnoidal fluid [8,9]. There are also previous reports in which the B-scan technique was used for this purpose [10,11]; however, these findings might be inaccurate as the B-scan method is not sensitive enough for measuring orbital structures [12,13]. As such, they may be clinically useful indicators of IH [14]. The ONH protrusion is mostly due to papilledema, which reflects swelling of the retinal ganglion cells that form the optic nerve (ON) due to axoplasmic stasis [15]. The mechanism of globe flattening (GF) is presumably mechanical [5,6,14], resulting from the imbalance between CSF pressure in the ONS and aqueous/vitreous humor pressure in the eye. Improved understanding of mechanical contributions to posterior GF in IH have implications for interpreting ophthalmic imaging changes as they might pertain to diagnosis and monitoring of IH and related conditions.

Numerical simulation is a useful tool to study the complex mechanical behavior of the ocular globe and study the quantities which are difficult or impossible to measure or manipulate experimentally. In the course of the last years, several numerical models have been developed based on the finite element method (FEM) to investigate on the effect of mechanical factors in the deformation of the eye globe and ONH head [16,17,18,19,20,21,22,23]. In this work, we performed a computational study of the eye globe response to ICP using FE analysis in order to look into the importance of the mechanical parameters contributing to posterior GF. The primary objective was to identify the roles of material properties of ocular tissues and the pressure loads. For this purpose, the GF was characterized and compared between Finite Element (FE) models and MR images from the patients with IH.

## 2. Materials and Methods

### 2.1. Model

**Figure 1:**
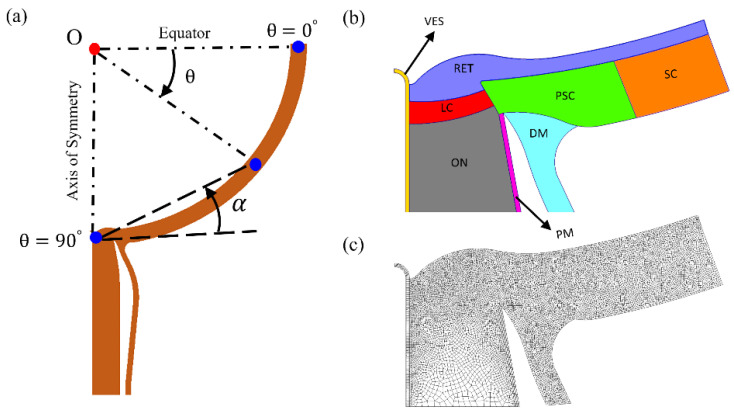
(**a**) Radially symmetric computational model of the posterior globe; the slope of each point with angle θ against the equator is denoted by angle α, (**b**) details of optic nerve head (ONH), and (**c**) the finite element mesh.

We constructed a generic axisymmetric two-dimensional (2D) model of the posterior globe and intra-orbital ON complex (Figure 1) based on the geometry and material properties of different regions of the globe and ONS complex reported in the literature [16,20,21,23]. In this two-dimensional model, with the ON/eye junction along the axis of radial symmetry, the sclera (SC) was modelled as a quadrant-spherical shell with a 12 mm internal radius and a thickness of 0.8 mm. The thickness of the retina (RET) was 0.27 mm. The lamina cribrosa (LC) was modeled centered on the ON complex, concentric to the scleral shell with an internal radius of 12.3 mm. The LC thickness at the axis of symmetry was 0.33 mm. The ON cup depth was 0.35 mm. The thickness of the peripapillary sclera was 0.48 mm at the canal wall. The wall thickness of the central retinal vessel (VES) was 0.05 mm. The dura mater (DM) and pia mater (PM), extending posterior to the globe, were assumed to be thicker at their scleral intersection, with the thickness of the DM tapered from 0.85 mm at the peripapillary sclera (PSC) to 0.36 mm at the posterior model boundary. Likewise, the PM thickness decreased from 0.12 to 0.06 mm over the same distance. The canal wall angle to the horizontal was taken to be 60^o^. The DM, ON, PM, and VES were extended 10 mm posteriorly from the SC. The space between the DM and PM, the SAS, holds the CSF.

ANSYS (Ansys, Inc., Canonsburg, PA, USA), GMSH [24], and FEniCS [25,26] softwares were used in order to create, mesh, and solve FE models. A non-uniform element density was used to generate FE meshes representing the generic eye geometry with two-dimensional axisymmetric plane elements. We increased the mesh density in a stepwise manner and refined the mesh to make sure that the results were mesh-independent. In the FE models, pressures were modeled as distributed loads acting perpendicular to the tissue surface exposed to the pressurized fluid. IOP was applied to the anterior surface of the RET and ICP to the SAS. The nodes on the axis of symmetry were constrained to remain on the symmetry axis and the nodes on the equator were constrained to deform radially [16,17,20,21]. The posterior ends of DM, PM, VES, and ON were fixed in all directions [17].

The mechanical response of the ocular tissues was represented by both linear and nonlinear material behavior. First, we applied linear elasticity theory as has been used in the literature in order to describe the biomechanical response of the ocular tissues [16,17,20,21]. We created 19,683 FE models consisting of all possible combinations of three levels (low, baseline, high) of linear elastic material properties for different domains (SC, PSC, LC, RET, DM, and ON) and three levels of the loading pressures, IOP and ICP, and the exact numerical values of these parameters are given in Table 1. The material properties of the VES were kept constant in order to simplify the analysis and limit the number of required FE models (preliminary studies showed that these domains have a limited effect on GF). We validated this linear elastic model against Sigal et al.’s work by plotting the maximum principal strain distribution [20] (results not shown).

Although the mechanical response of ocular tissues has been studied based on the theory of linear elasticity for simplicity, their actual biomechanical response is nonlinear and anisotropic [27]. Therefore, in separate models, we represented the mechanical response of the SC, PSC, PM, and DM as a nearly incompressible neo-Hookean material [28], while the ON, LC, VES, and RET were kept as linear elastic (Table 2). The strain energy function *W* for nearly incompressible hyperelastic materials is given by Equation (1):

(1) $$W=c_{1}\left({\tilde{I}}_{1}-3\right)+\frac{K}{2}{\left[Ln\left(J\right)\right]}^{2}$$

where ${\tilde{I}}_{1}$ is the first invariant of the isochoric part of the right Cauchy-Green deformation tensor, *J* is the determinant of the deformation gradient, $c_{1}$ is the material constant, and *K* is the bulk modulus. The FE implementation of the nearly incompressible neo-Hookean model in FEniCS was validated against the analytical solution of a single finite element (results not shown). The material parameters were chosen near the range of the values published in a previous study [23] and a second set of 19,683 nonlinear models was solved by letting the material parameters and pressure loads take their low, baseline, and high values, as given in Table 2. The bulk modulus and the Poisson’s ratio were chosen to be 0.05 GPa and 0.49 for all hyperelastic and linear elastic domains, respectively.

### 2.2. Assessment of Globe Flattening and Model Comparison

**Figure 2:**
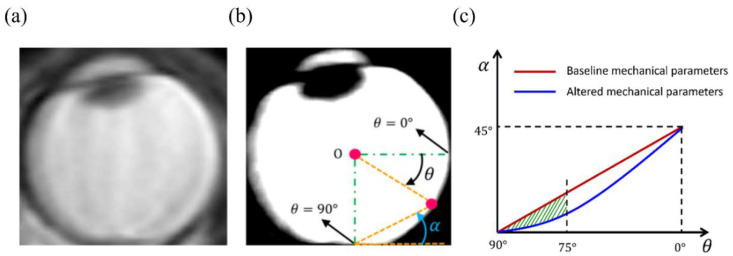
(**a**) An MR scan of the orbit in a patient with high ICP done prior to treatment for high ICP. (**b**) The same MR image after quality enhancement. The parameters of angle-slope analysis are also shown. (**c**) ω is defined as the area between angle-slope curves corresponding to numerical models created using baseline and altered mechanical parameters.

We developed an angle-slope analysis for quantifying the GF. To this end, all peripheral points on the inner retinal surface of numerical models after deformation were represented by the angle θ that they make with the equator line. Furthermore, α was defined as the angle that a given peripheral point, defined by θ, makes with respect to the horizontal line passing through the point representing θ = 90°, at the back of the globes. Since the angle α is small and almost zero where GF exists, plotting the variation of slope (α) versus angle (θ) can be used to detect the region with GF (Figure 2).

We defined a new measure, ω, for quantifying the amount of GF in different numerical models that is defined as area between the angle-slope curve for a numerical model created using baseline values (note that this curve is an almost straight line with a constant slope) and the angle-slope line for any other model created using different combinations of parameters (Figure 2). The measure ω was calculated from 75° ≤ θ ≤ 90° to simplify the calculation and make the measure more relevant to our clinical observations that GF seldom observed beyond θ ≅ 75°. Any non-zero positive value of ω indicates some levels of convexity loss in the globe; thus, this measure can be used to detect and represent the GF. Furthermore, because ω increases strictly with increasing the flattening area, this measure can be used to evaluate the sensitivity of the GF to the numerical model parameters.

In order to evaluate the level of contribution of the parameters to the GF, we sorted the FE model solutions within each set (linear and non-linear) from the highest to the lowest ω and defined the top 10% as “high GF models”. Almost identical results were obtained for analysis of the top 5% of models, sorted by ω (results not shown). We examined the prevalence of parameter values (low, baseline, and high) in the high GF models for each model set to identify which parameters had the greatest impact on GF. Because IOP is known not to be a factor in globe flattening, this analysis was stratified between IOP levels.

### 2.3. Tuning of Models to Human Images

We used clinical observations (*n* = 5) in order to assess the ability of the numerical model to represent GF observed in patients with IH prior to treatment. Selection of patients with high-resolution MR imaging was done retrospectively based on review of patients seen in the neuro-ophthalmology division at Byers Eye Institute at Stanford. This work followed the tenets of the Declaration of Helsinki and was approved by an institutional review board at Stanford University with a waiver of informed consent (protocol number: 50,927 and FWA00000935). Mid-axial high-resolution Fast Imaging Employing Steady-state Acquisition (FIESTA) or T2 MR images at the level of the ONH were identified and were used to characterize the amount of posterior eye GF (Figure 2). First, the center of the globe was found and defined as the origin of polar coordinate system (point O). In this coordinate system, all peripheral points on the inner retinal surface were represented by the angle θ that they make with the equator line. The angle-slope analysis technique, as described above, was applied to detect and quantify GF.

## 3. Results

**Figure 3:**
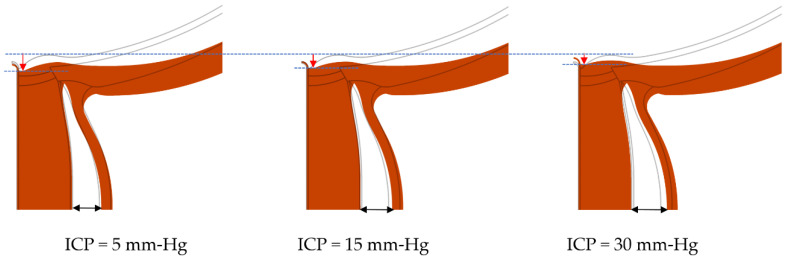
Flattening of the globe and distension of the ONS with increasing the ICP. The grey black lines correspond to the undeformed state and the deformations are 5× exaggerated. Vertical red arrows demonstrate magnitude of deformation at the back of the globes. The dashed lines are included to facilitate comparison. The two-sided arrows show the widening of the ONS as ICP increases. For all models shown, IOP = 15 mm-Hg and the Young’s modulus of SC, PSC, LC, RET, DM, ON, PM, and RET was chosen to be 0.6, 0.6, 0.3, 0.03, 1.8, 0.06, 1.2, and 0.03 MPa, respectively.

Figure 3 illustrates geometric model solutions for increasing levels of ICP with other model parameters held constant. In addition to a dose-response effect on globe flattening, as demonstrated by increasing length of red vertical arrows, there is accompanying widening of the ONS, as demonstrated by increasing length of black two-sided arrows. This distension of ONS is another sign seen on MR images of people with IIH [6,29,30,31] and some astronauts [32,33].

**Figure 4:**
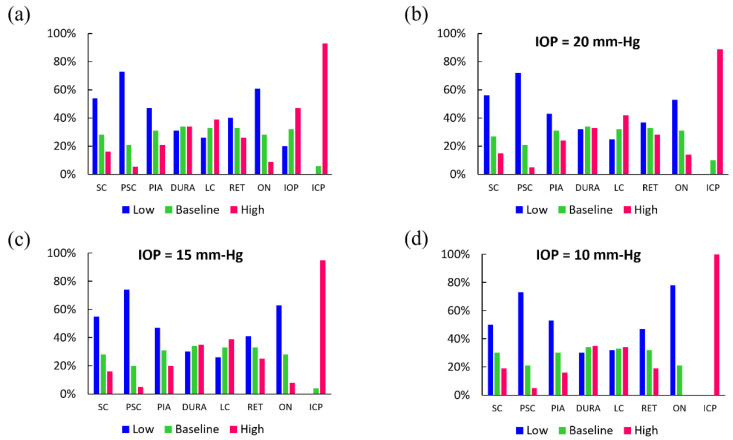
(**a**) Distribution of mechanical parameters (taking their high, baseline, and low values) in linear models with highest decile of GF, i.e., numerical models that showed significant GF in FE models where all tissues were modelled as linear elastic materials (Table 1). Distribution of mechanical factors in GF models when IOP was (**b**) 20 mm-Hg, (**c**) 15 mm-Hg, (**d**) 10 mm-Hg.

The 19,683 linear FE models, varying by material properties and pressure magnitude, were sorted based on the amount of GF that is quantified by the measure ω. Figure 4a is a histogram graph showing the distribution of each parameter’s level (low, baseline, high) in the linear elastic FE models with upper decile of GF (high GF models), i.e. those with $44\%<\bar{\omega }<100\%$. Here, $\bar{\omega }$ = ω/ω _max_ × 100 and ω _max_ is the maximum area calculated between the angle-slope curves of all FE models. The largest amount of GF primarily occurred in linear FE models with low Young’s modulus for the ocular tissues, especially that of the ON, SC, and PSC tissues. About 54% and 72% of high GF models had low scleral Young’s modulus and low peripapillary scleral Young’s modulus, respectively. More than 90% of models had high ICP and none had low ICP. The ON and PIA were also recognized as being important.

It is known that the IOP of patients with IH is not typically elevated. Thus, we stratified high GF models according to IOP level (high, baseline, or low) (Figure 4b–d). IOP at the baseline value of 15 mm-Hg aligns with the typical clinical case where IOP of patients with IH is not elevated (Figure 4c). It is seen that independent of the IOP value, the majority of high GF models had sclera and peripapillary sclera with a low Young’s modulus. Interestingly, high ICP became a more prevalent factor in high GF models as IOP decreased, i.e., about 89%, 95%, and 100% of high GF models had ICP = 30 mm-Hg when IOP was equal to 20, 15, and 10 mm-Hg, respectively. This suggests that high IOP shields the effect of high ICP on GF to some extent.

**Figure 5:**
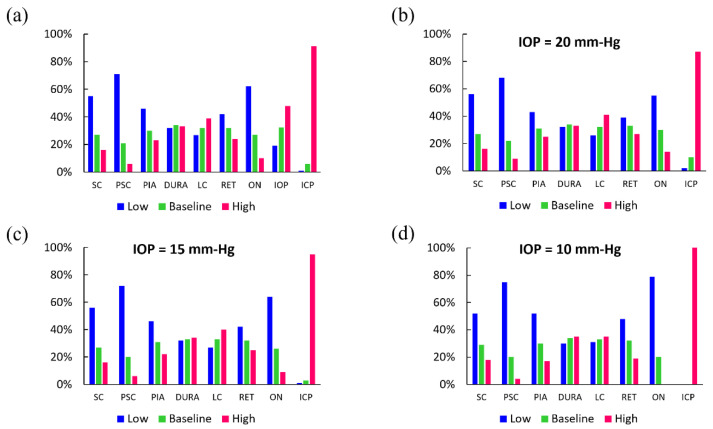
(**a**) Distribution of mechanical parameters (taking their high, baseline, and low values) in mixed material models with highest decile of GF, i.e., numerical models that showed significant GF in FE models where some tissues were modelled as nonlinear materials (Table 2). Distribution of mechanical factors in GF models when IOP was (**b**) 20 mm-Hg, (**c**) 15 mm-Hg, (**d**) 10 mm-Hg.

A similar analysis was done for values of the measure ω that were obtained from 19,683 mixed linear and nonlinear FE models. It is again observed that the largest amount of GF primarily occurred in FE models with ocular tissues often having the low-value material properties (Figure 5a). The nonlinear models gave almost the same result for the importance of the elevated ICP in the GF, i.e., 91% of high GF models had high ICP and decreasing the IOP to 10 mm-Hg showed that 100% of high GF models had ICP = 30 mm-Hg (Figure 5a–d).

**Figure 6:**
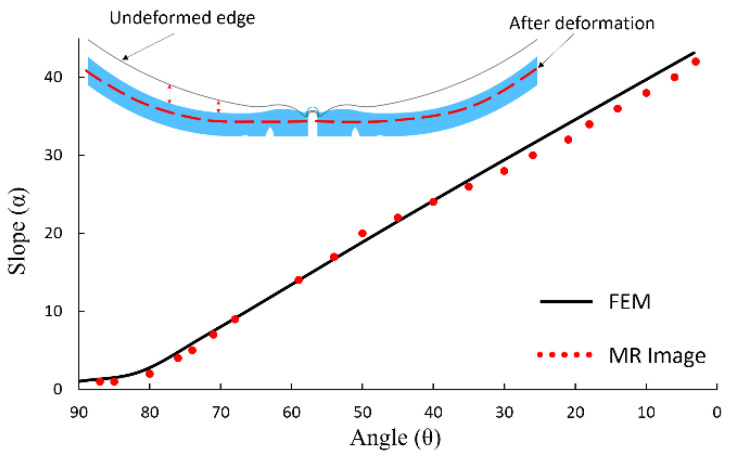
Comparison between the numerical FE model and MR image of a patient with IIH (shown in Figure 2) using the angle-slope analysis. The inset shows the flattened and normal globe obtained from the numerical model of the posterior globe. The two-sided arrows show the area where loss of convexity (i.e., GF) occurred at the posterior of the globe. In the FE model, IOP = 15 mm-Hg, ICP = 30 mm-Hg, and Young’s modulus of SC, PSC, LC, RET, DM, ON, PM, and RET was 0.6, 0.6, 0.3, 0.03, 1.8, 0.06, 1.2, and 0.03 MPa, respectively. These model parameters were used in Figure 3 for investigating the influence of ICP on GF and ONS distension.

The angle-slope technique was implemented on MR images of people with IH in order to quantify the amount of GF. Figure 2 shows a typical MR image with obvious globe flattening. The parameters, i.e., the pressures and material properties, of the numerical model were altered in order to determine whether the same angle-slope behavior could be obtained numerically. We took advantage of our parametric study and only altered the most important factors with the range that is given in Table 1. This allowed us to limit the number of trials required for numerically matching the angle-slope curves of MR images. The angle-slope variations of a typical MR image (Figure 2) and its corresponding numerical model are shown in Figure 6. Similar agreement was seen for MR images of the other four cases and we were able to perform the same analysis using material parameters of Table 2 (results not shown). The generic FE model for each case had a slightly different set of model parameters; however, ICP (about 30 mm-Hg) was always significantly higher than its baseline level, and SC, PSC, and ON Young’s moduli were always close to their low values. The exact calibration of model parameters in an inverse model requires careful attention and is beyond the scope of the present work.

## 4. Discussion

Elevated ICP has been suggested as a causative factor for flattening of the posterior ophthalmic globes seen clinically in IIH and other high ICP situations [2,3,4,14,34,35]; however, there is no previous study attempting to explain this phenomenon in terms of a purely mechanical mechanism. The primary goal of this study was to determine mechanical factors contributing to the posterior eye globe flattening using a numerical study. Not only do numerical models allow the isolation of mechanical from biological processes, but they also provide the opportunity to manipulate model parameters and measure their outputs. Both of these features are not easily accomplished experimentally. In the present study, we performed a parametric study of posterior ophthalmic globe mechanics using generic geometry under pressure loads inside the globe (i.e., IOP) and ONS (i.e., ICP). Advances over previously published models of the ON globe complex include focus on GF and ICP as well as use of 2D axisymmetric, nearly incompressible neo-Hookean hyperelastic models in four subdomains of SC, PSC, DM, and PM, while the other tissues were assumed to be linear elastic. We introduced a measure to quantitatively assess the GF. Furthermore, we developed and applied the angle-slope technique to characterize and compare the GF in the numerical models. Through manipulation of material properties and pressures, we achieved a very good agreement between the numerical results and MR image analyses of patients with IH. Thus, the proposed FE model may be used in future studies to perform inverse modeling based on MR images of patients with IH in order to gain insight into the basis of variation in GF between individuals.

The histogram graphs, shown in Figure 4 and Figure 5, contain important statistical information about the role of model parameters in GF. In general, the elevated ICP had the most important role. The sclera, posterior sclera, and ON stiffness also played a role in GF in both linear elastic models and mixed linear/nonlinear elastic models, i.e., decreasing the elastic modulus of SC, PSC, and ON increased the flattening of the posterior globe (Figure 4). IOP was manipulated in the initial model comparison for full exploration of the parameter space. The stratified analysis further highlights the contribution of factors to GF by observing that ICP was a necessary mechanical factor contributing to the deformation of the globe, particularly when IOP is not elevated, as is typically the case in IIH. The importance of ICP was also demonstrated by the mixed linear/nonlinear FE models (Figure 5). Figure 5 clearly demonstrates the obvious role of the increased ICP in GF, where 91% of the models had ICP at the elevated level (e.g., 30 mm-Hg). In other words, high ICP is the most important mechanical factor in GF. We note here that the distributions of mechanical factors were fairly similar between the mixed linear/nonlinear and fully linear elastic models.

The finding of scleral stiffness having a large influence on the mechanical response of the back of the eye globe to external loads is in agreement with the previous finite element modeling simulations demonstrating that SC and PSC are the most important load-bearing connective tissues in the posterior eye [20]. The ON stiffness was also an important parameter in GF. Figure 4a showed that ON had its lowest Young’s modulus in about 61% of all models with high GF. We also observed that when IOP = 10, 15, and 20 mm-Hg, about 78%, 63%, and 53% of high GF models had ON tissue with low Young’s modulus, respectively; thus, the influence of ON material properties on GF is dependent on IOP. The stiffness of DM and LC did not seem to play a prominent role in GF. These findings are in general agreement with previous studies on the importance of material properties of various ocular tissues in biomechanical response of the ONH as well as their roles in altered ICP-related diseases [16,20,23].

The angle-slope technique for quantifying GF was successfully implemented on MR images from patients with IH. Taking an inverse approach, we were able to tune the finite element model by altering the parameters to match with these image processing results. For this purpose, we used the results of our parametric study and varied model parameters according to their importance in GF. We stayed within the range of parameter values given in Table 1 in the process of tuning the numerical model. This demonstrates the relevance of our numerical approach for investigating GF in patients. It should be noted that the degree of GF in MR images of different patients was not the same (results not shown), and that this manifested itself as different material properties in the FE model. This suggests that one explanation for differences in GF between patients is different material properties of ocular structures. However, it is noted that this analysis is preliminary and does not consider neither the complexity in the 3D geometry of ocular tissues nor their nonlinear inhomogeneous material properties. Thus, we include it here to demonstrate the feasibility of using FE models to perform inverse modeling based on MR images of patients with IH in order to gain insight into the possible changes in the material properties of ocular tissues of these patients contributing to the GF and variation in the GF between individuals.

This work has certain limitations. First of all, the present work is based on a simplified 2D axisymmetric generic model of the eye globe [19]. The primary advantage of generic models is their capability for conducting parametric analysis because of their lower computational cost compared to three-dimensional (3D) models. They could be used to create many different models by altering the parameters of interest in a systematic way and subsequently identify the most influential biomechanical factors [16,17,19,20,23,36]. The main drawback of 2D models [16,17,37] is that they do not reflect the 3D complex geometry of the ocular globe [18,19]. We are currently working on developing a 3D model of the eye globe to extend this study by constructing individual-specific models of human ON, like what has been done in another study [22]. Another limitation of the present study is that all materials were assumed to be linear elastic, isotropic, and incompressible without considering collagen remodeling and nonlinear mechanical response of ocular tissues. In order to address this limitation to some degree, we also included the numerical FE models in which a nonlinear hyperelastic material model was used to represent the mechanical response of a number of ocular tissues. However, it is noted that anisotropic material models are needed for modeling the mechanical behavior of ONH and neo-Hookean solids are only suitable for first-pass efforts [28]. This is because collagen fibers are expected to play a role in the mechanical response of sclera and peripapillary sclera [27,38,39,40]. It is noted that three-dimensional models are required to be able to consider anisotropic material models [27,41], and such models will limit the number of models that can be solved because they are computationally expensive. In the current work, possible roles of parameters such as extraocular muscles, orbit fat, and choroidal vasculature were not considered. The numerical models did not include choroid, as has been included in a recent study [42]. The uniqueness and goodness of model parameters are topics of future work and were not covered in this work. Optimization techniques can be used to confirm the possible uniqueness of model parameters [43]. If different parameter sets are found to match the reported model results to the MR images of the patients, additional information, e.g., clinical/experimental measurements of mechanical factors, are needed to guide the inverse modeling procedure. In spite of these limitations, the present study successfully demonstrated how pressures and material properties of ocular tissue interact to cause shape changes of the posterior globe. Furthermore, although this work did not create a finite element model based on features that are measurable in the clinic, it showed the possibility of using a numerical model to capture the GF observed in patients with IH.

In summary, we investigated the dependence of posterior globe flattening on material properties of ocular tissue and pressure loads in this numerical study. We observed that elevation of ICP would lead to a higher level of flattening. Identification of the elevated ICP as an important mechanical factor in the GF is in agreement with experimental studies [4,14,15,35]. Furthermore, our results suggest that reduction in ON and scleral stiffness pre-disposes to this deformation. These deformations could be of clinical relevance. For example, elevated ICP is the underlying cause of ophthalmic morbidity in IH and measurements of the posterior globe have been demonstrated to change in association with ICP level and ICP treatment [2,4,14,44]. Numerical models such as the present one could guide interpretation of clinically observed globe changes in IH states.

## Figures and Tables

**Table 1:** The range of pressures and material properties of different domains in linear elastic numerical models. The Poisson’s ratio is 0.49 for all domains.

Parameters	Abbreviation	Unit	Value		
			Low	Baseline	High
**Pressure**					
**Intraocular pressure**	IOP	mm-Hg	10	15	20
**Intracranial pressure**	ICP	mm-Hg	5	15	30
**Young’s modulus (E)**					
**Sclera**	SC	MPa	0.6	1.2	1.8
**Peripapillary sclera**	PSC	MPa	0.6	1.2	1.8
**Pia mater**	PM	MPa	0.6	1.2	1.8
**Dura mater**	DM	MPa	0.6	1.2	1.8
**Lamina cribrosa**	LC	MPa	0.3	0.6	0.9
**Retina**	RET	MPa	0.03	0.06	0.09
**Optic nerve**	ON	MPa	0.03	0.06	0.09
**Retinal vessel**	VES	MPa		0.1	

**Table 2:** The range of pressures and material properties of different domains in mixed linear and nonlinear elastic numerical models.

Parameters	Abbreviation	Unit	Value		
			Low	Baseline	High
**Pressure**					
**Intraocular pressure**	IOP	mm-Hg	10	15	20
**Intracranial pressure**	ICP	mm-Hg	5	15	30
**Nonlinear material constant (*c*_1_)**					
**Sclera**	SC	MPa	0.1	0.2	0.3
**Peripapillary Sclera**	PSC	MPa	0.1	0.2	0.3
**Pia Mater**	PM	MPa	0.1	0.2	0.3
**Dura Mater**	DM	MPa	0.1	0.2	0.3
**Young’s modulus (E)**					
**Lamina Cribrosa**	LC	MPa	0.3	0.6	0.9
**Optic Nerve**	ON	MPa	0.03	0.06	0.09
**Retina**	RT	MPa	0.03	0.06	0.09
**Vessel**	VES	MPa		0.1	
